# A Study on Online Intervention for Early Childhood Eating Disorders during COVID-19

**DOI:** 10.3390/ijerph19063696

**Published:** 2022-03-20

**Authors:** Silvia Cimino, Carlos A. Almenara, Luca Cerniglia

**Affiliations:** 1 Department of Dynamic, Clinical, and Health Psychology, Sapienza, University of Rome, Via Degli Apuli, 1, 00186 Rome, Italy; silvia.cimino@uniroma1.it; 2 Faculty of Psychology, Universidad Peruana de Ciencias Aplicadas, Av. Alameda San Marcos, Lima 11-15067, Peru; carlos.almenara@upc.pe; 3 Faculty of Psychology, International Telematic University Uninettuno, Corso Vittorio Emanuele II, 39, 00186 Rome, Italy

**Keywords:** children, disordered eating, online intervention

## Abstract

Eating disorders are among the most common clinical manifestations in children, and they are frequently connected with maternal psychopathological risk, internalizing/externalizing problems in children, and poor quality of mother–child feeding exchanges. During the COVID-19 lockdown, in person assessment and intervention were impeded due to the indications of maintaining interpersonal distancing and by limits to travel. Therefore, web-based methods were adopted to meet patients’ needs. In this study N = 278 participants completed the SCL-90/R and the CBCL to examine the psychopathological symptoms of mothers and children (age of the children = 24 months); moreover, the dyads were video-recorded during feeding and followed an online video-feedback based intervention. Maternal emotional state, interactive conflict, food refusal in children, and dyadic affective state all improved considerably, as did offspring internalizing/externalizing problems and mothers’ depression, anxiety, and obsession–compulsion symptoms. This study showed that video-feedback web-based intervention might be employed successfully to yield considerable beneficial effects.

## 1. Introduction

This study proposes the results of a sustainable strategy for the treatment of eating difficulties in early childhood. It is now widely demonstrated that advances in technology significantly facilitate the data collection and intervention processes [1]. Online-based strategies for the assessment and follow-up of patients can be performed proficiently, with very little need for subjects to be present for on-site evaluations. Moreover, online observation of children and their significant relationships has proven to be an effective method of clinical evaluation for obtaining comprehensive information on children’s affective, cognitive and social self-organizational characteristics [2].

This study intends to address the current and very common difficulties of families with children with disordered eating to access assessment and intervention contexts due to economical and geographical problems, and to the impossibility of encountering a professional face-to-face given the restrictions imposed during the COVID-19 pandemic. To achieve this goal, an observational measure for the evaluation of the feeding difficulties [3] is proposed for online use.

### 1.1. Why Focusing on Disordered Eating in Early Childhood?

Eating difficulties are among the most frequent clinical manifestations in childhood [4]. The scientific literature configures childhood eating disorders according to two general dimensions: on the one hand, the focus is on the behavioral manifestations of the child who despite the availability of food does not ingest an age-appropriate amount of nourishment or ingests too much of it, on the other hand, it focuses on the possible maladaptive aspects of the caregiver–child relationship, which tend to manifest in these clinical pictures [5]. Child’s feeding problems can take on multiple clinical manifestations, from transient selectivity for certain foods to complete and prolonged rejection of any food [6].

These problems may manifest as eating disorders (ED) or as sub-threshold problems, which are frequently overlooked both by researchers and clinicians.

A lack of appetite, little interest in eating, lower satisfaction during a meal, the recurrent search for the same selected items, and the refusal of food in circumstances of frustration were among the sub-threshold feeding symptoms. Indeed, sub-clinical eating problems are widespread in children; in fact, youths with typical development have a disordered eating prevalence of around 25%, whereas children with developmental problems have a prevalence of 80% [7,8]. According to the Developmental Psychopathology clinical and theoretical framework [9], the risk of developing disordered eating in children is mediated and moderated by the influence of the interactions between the individual (e.g., temperament, emotional intelligence, etc.), genetic predisposition and the maladaptive, negative or stressful environmental experiences, particularly in the family environment, with the exposure to parental psychopathological risk [10]. A large bulk of literature has demonstrated that the quality of interactions between caregivers and their children with eating problems is frequently poor, with low-quality exchanges during feeding and play [11]. Parent–child relationships have been found to be characterized by caregivers’ intrusiveness or withdrawn in interactive interactions with their children, due in part to dysfunction in the neurobiological reward circuits [12]. These maladaptive relationship patterns, which may have a hereditary base, can appear as early as the first year of life, and are major contributors to the emergence of childhood eating disorders [13]. On the other hand, parents’ attention and sensitivity to the needs of their children, such as the identification of hunger and satiety cues from nursing and weaning, have been recognized as an important protective factor from the onset of early disordered eating [14,15,16].

### 1.2. The Impact of COVID-19 on Research: Difficulties and Opportunities

The worldwide spreading of the COVID-19 pandemic has strongly influenced the lives and habits of most people, modifying interpersonal interactions, work and school routines, and the possibility of administering research and clinical assessments in person, in the house of families with children with disordered eating [17]. On the other hand, even before the pandemic outbreak, the possibility of administering tools and clinical sessions online had been proposed and largely used, with mixed but overall positive results [18,19]. Technology-mediated assessment and intervention programs have allowed exciting new opportunities to study processes related to the development, maintenance, treatment, and long-term course of eating disorders. E-Health, offers the tremendous opportunity to expand equity and equal delivery and funding for mental health services [20,21]. At present, where rising (mental) healthcare spending is a problem, the potentially lower cost of e-health is especially enticing. E-health and the usage of Information and Communication Technologies (ICT) to facilitate or improve health care have drawn the attention of policymakers and health decision makers who believe that the frequent use of e-health technologies can contribute to more independent, effective, efficient and high-quality care for patients [22,23]. This would in turn encourage e-health advances in prevention to treatment strategies. In times when face-to-face treatments are encountering severe limitations due to the COVID-19 pandemic, e-health assessment and intervention can greatly help health care delivery approaches.

Moreover, recent studies demonstrated that e-health can benefit on the environment by promoting remote health programs that reduce CO_2_ emissions since patients no longer have to travel to their health facility [24,25,26,27]. In the current climate of growing interest in reducing the environmental impact of health care activities [28,29,30,31], there is an urgent need of actions aimed at further evaluating the impact of telemedicine programs for reducing the environmental footprint of (mental) healthcare procedures.

### 1.3. The Present Study

Recently, the Horizon Program 2020 encouraged researchers and professionals to test web-based psycho-educational intervention strategies for parents and families, as well as the use of technology-mediated systems to prevent and/or intervene on psychopathology, particularly in the case of children’s depressive and anxiety symptoms, and feeding difficulties. Early childhood psychological prevention and intervention foster mutually attuned and sharing sensitive parent–child relationships during feeding, which is critical to the child’s learning of adaptive behavioral and emotional regulation [32]. As a result, researchers and clinicians are being urged to focus on evaluation, prevention, and intervention, including the use of technology-mediated tools, in order to promote parenting of young children and to minimize the likelihood of maladaptive consequences.

Based on the above considerations, the present study (performed during the COVID-19 lockdown) evaluated longitudinally, over two assessment points, the effect of a web-based intervention aimed at improving the quality of mother–child feeding interactions. Our hypotheses were that the video-feedback treatment could improve mother–child interactions during mealtimes and diminish mothers’ and children’s psychopathological symptoms.

## 2. Materials and Methods

### 2.1. Participants

A total of 342 mothers and their children with disordered eating (restrictive sub-threshold clinical manifestation reported by psychiatrists and psychologists based on the Zero-to-Five diagnostic classification [33] were recruited during the COVID-19 lockdown (November 2020), in collaboration with public mental health centers in northern, central and southern Italy. Mothers were contacted by psychologists who informed them about the aims and procedure of the study. A convenience sampling method was employed [34] with a simple random sampling method to draw names of the children and their parents, using the existing lists at the centers [35].

Subjects who wanted to take part in the study signed a written informed consent and were instructed on how to fill out the questionnaires. Following the Declaration of Helsinki, the Ethical Committee of the Psychology Faculty at Sapienza University of Rome accepted the study before its commencement (Protocol Number: 0000809).

The inclusion criteria were: infants aged 24 months; mothers and/or children without other physical or psychological disorders except disordered eating in children. We did not include families in which the children and/or mothers were receiving pharmacological or psychiatric interventions (N = 11), families in which the children had physical or psychological problems (N = 9), or families in which the parents referred existing or prior psychological conditions (N = 12), families in which the mothers did not complete all of the questionnaires (N = 21), and families in which the mothers did not complete all of the measures (N = 11).

As a result, the final study sample included N = 278 families of children aged 24 months (144 males and 134 females).

### 2.2. Procedure

Although the recruitment was performed with the help of mental health centers, all procedures took place remotely through online surveys and remote observation, according to COVID-19 pandemic restrictions and protocols.

At T1 and T2 (after four weeks, during which the intervention took place remotely twice a week with one-hour sessions; see below for details), mothers completed the Symptom Checklist-90-Revised (SCL-90-R) [36,37], a self-report measure for assessing their own psychopathological symptoms. They also filled out the Child Behavior Checklist (CBCL 1½–5) [38], for assessing children’s emotional–behavioral functioning. Mother–child feeding interactions were remotely video-recorded (20-min videos) following a validated method described below (Scala di Valutazione delle Interazioni Alimentari, SVIA; Lucarelli et al. [39]. The feeding interactions were coded by two separate raters (Cohen’s k = 0.89) who had been qualified to use the instrument in general and clinical populations. The overall agreement with expert encoders for durability is between 0.87 and 0.92.

The intervention model for the psychological support to the parent–child relationship was based on the SVIA video recording and followed the Video-Intervention Therapy-VIT approach described by Stein et al. [40,41] for samples with disordered eating in childhood. The Feeding Scale and its Italian version (SVIA—developed by our group with Prof. Chatoor’s supervision) [3] has been used in this field to examine caregiver–children’s at-risk patterns of interactions that can be linked to children’s inadequate capacity to regulate their emotions and behavior, which is commonly linked to eating disorders [42,43,44,45]. During feeding, a negative maternal affective state and the presence of interactions characterized by conflictual, non-collaborative, and non-empathetic communication may contribute to children’s food refusal and a general negative emotional climate, making feeding an unpleasant experience for both the mother and the child, contributing to the creation of a vicious cycle in which maladaptive patterns in the dyad are perpetuated. After utilizing this measure in daily-life settings (a method that increases ecological validity), for more than two decades [46,47,48], our group began to test it in remote administration, with positive results.

In the present study the intervention took place in an online platform, and (in a subsequent session) the clinicians observed together with the mother some specifically selected sequences of video recordings of mealtime, supporting the caregiver in recognizing the relational signals of their child. During online sessions, the therapist remotely recorded the mother and infant at home during mealtimes; then, the therapist and mother viewed and discussed snippets chosen by the therapist to emphasize the infant’s signals and exploration, as well as to draw out and strengthen the mother’s observational abilities. The treatment was three-fold. First, it focused on the infant’s point of view, concentrating on his or her signs (initiatives toward the mother, exploratory behavior, indications of hunger and satiety, and attempts at self-feeding). The mother’s perspective was included in the second stage, which highlighted mother–baby efforts, joint responses, shared emotional experiences, and the success of sensitive, quick answers to infant signs. Third, the videotapes were utilized to assist the mother in identifying and addressing prospective mealtime conflict triggers.

### 2.3. Measures

The CBCL 1½–5 [38] consists of a report–form tool composed of 99-item tapping children’s emotional/behavioral problems. Parents are asked to report their child’s: Internalizing Problems (which includes the scores of Emotionally Reactive, Anxious/Depressed, Somatic Complaints, Withdrawn syndrome scales) and Externalizing Problems (which includes scores of Attention Problems and Aggressive Behavior syndrome scales). This measure is widely used in diverse studies to evaluate childhood emotional and behavioral symptoms (see for instance [49].

The SCL-90-R [36] is a 90-item self-report questionnaire tapping psychological symptoms and psychological distress in adults on a Likert scale ranging from 0 (not at all) to 4 (extremely). This measure is commonly used for screening and evaluation of psychological symptoms in adults of both clinical and the general population. The tool is composed of nine dimensions: Somatization, Obsessive–Compulsivity, Interpersonal Sensitivity, Depression, Anxiety, Hostility, Phobic Anxiety, Paranoid Ideation, and Psychoticism, and of a Global Severity Index (GSI). The Italian validation [37] showed good reliability (Cronbach’s α = 0.70–0.96).

The SVIA [48] is the Italian adaptation of the Feeding Scale [3]; it can be used with dyads with 12–36 month old children. It evaluates the quality of the parent–child interaction during feeding. Parent–infant interactions are video recorded for at least 20 min, and then a series of behaviors and emotional states are coded and evaluated. This tool consists of 41 items that form four subscales: (1) Parent’s affective states; (2) Interactive conflict; (3) Food refusal behavior; and (4) Dyad’s affective state. Higher scores on each scale refer to greater relational difficulties. The SVIA showed good reliability in terms of internal consistency (Cronbach’s α = 0.79–0.96).

### 2.4. Statistical Analyses

The scores at T1 and T2 (before and after the intervention) on all measures were compared using analyses of variance (ANOVAs) for repeated measures. The calculated *p* values are reported, with values <0.05 being accepted as significant. Mean values are reported with SDs. A power analysis was conducted accordingly to Cohen’s (2013) [50] suggestions, α was set at 0.05 and a power of 0.862 was obtained with a large effect size of (f^2^ = 0.46) (a large effect size indicates that the effect is powerful in the explanation of single events). All analyses were performed using IBM SPSS Statistics software for Windows, Version 25.0 (IBM Corporation, Armonk, NY, USA).

## 3. Results

The mean age of the children was 24.2 months (SD = 2.41). The mothers’ mean age was 34.17 (SD = 2.31). Most of the subjects were Caucasian (91%), with the majority of mothers having completed high school or university (88%); 95% of mothers were married, and all households were of average socioeconomic status (94% had an annual income of 25,000–30,000 Euros). All mothers reported high satisfaction and comfort with telehealth and ICT responding to the Telehealth Satisfaction Scale (TeSS; Goodman et al. [51]).

An ANOVA revealed main effects of time point (all *p* < 0.001) on all four SVIA subscale scores. Bonferroni’s post hoc tests demonstrated that SVIA scores at T2 were significantly lower (i.e., less maladaptive) than T1 for all four subscales: mother’s affective state; interactive conflict; food refusal; dyad’s affective state. The mothers’ average scores for each SVIA subscale at T1 and T2, and η^2^ values are reported in Table 1 (Eta squared is a measure of the effect size).

An ANOVA of the SCL-90/R subscales and GSI scores for mothers across time points revealed a significant main effect of time point (*p* < 0.001). GSI scores were significantly lower at T2. In particular, mothers showed high scores in the subscales of Depression, Anxiety and Obsessive–Compulsion. Table 2 shows means and η^2^ values.

Children’s emotional/behavioral functioning was rated by mothers as less maladaptive at T2, especially in the subscales of Withdrawn, Anxious/Depressed, and Aggressive Behavior. Children also showed significantly lower scores in the Internalizing and Externalizing subscales. Table 3 shows means and η^2^ values.

## 4. Discussion

This study presented results of the online application of a treatment of low-quality parent–child interactions and children’s disordered eating. Our hypothesis was that the video-feedback treatment could improve mother–child interactions during mealtimes and diminish mothers’ and children’s psychopathological symptoms.

The results confirmed our hypotheses by showing that the online intervention was effective at increasing the quality of mother–child interactions during feeding. In fact, SVIA scores at T2 were more adaptive than T1; in particular, scores on all four subscales of the SVIA improved (mother’s affective state, interactive conflict, food refusal, dyad’s affective state). Notably, the improvement of the quality of mother–child feeding interactions was very significant. Previous literature assessing the effectiveness of video-feedback in non-web-based treatments (see for example [52]) also showed high rates of success in the treatment of feeding difficulties. In this sense, our results are comparable to in-person intervention, although in this study we did not recruit a control sample to statistically compare face-to-face treatment and remote intervention. Even if video-feedback intervention to encourage positive parenting is a consolidated method [53,54], to the best of our knowledge no study has evaluated the effectiveness of such an intervention online. If our results are confirmed in future research, it would be a very significant achievement. In fact, clinicians and patients could benefit from both an effective psychoanalytically oriented strategy and from the value of technology, which allows a reduction in costs in reaching out to all potential individuals in need.

Moreover, online intervention was able to reduce psychopathological symptoms of the mothers, both in terms of global risk (SCL-90/R Global Severity Index) and with regards to specific subscales. Specifically, at T2 maternal scores of Depression, Anxiety and Obsessive–Compulsion were significantly lower. A large bulk of previous literature demonstrated that depression and anxiety symptoms in mothers are associated with feeding disorders in offspring and are predictive of maladaptive patterns of parent–child interactions [55,56]. Obsessive–compulsive maternal symptoms are given less attention in this field, although recently some studies focused on this issue finding weak or mild links with feeding difficulties in children [57,58,59]. These studies specifically stressed the role of mother’s perceptions, repetitive negative thoughts, and obsession about her child’s eating, which can worsen their eating problems. In line with this literature, we assume that maternal symptoms can be considered both as outcomes of the low-quality relationship and as a predictor of poor interactions [60]. Therefore, we cannot draw conclusions from this result. We can only observe that psychopathological symptoms were reduced, particularly in three important areas such as depression, anxiety and obsessive–compulsion. It is also noteworthy that this study took place during the COVID-19 lockdown. Given the huge complexity of this environmental event, we cannot rule out the possibility that the change in the SCL scores were linked to the pandemic situation as suggested by previous research [61].

As per the children’s emotional/behavioral functioning, at T2 the scores at Withdrawn, Anxious/Depressed, and Aggressive Behavior subscales significantly decreased. Further, children showed significantly lower scores in the Internalizing and Externalizing subscales. This result is consistent with previous literature that posited that the low quality of parent–child interactions is associated with the child’s reduced capacity to cope with distress and to regulate their emotional/behavioral states [62]. Thus, we propose that our online treatment reduced psychopathological symptoms in children, owing to the improvement in mother–infant relationship. Of course, this speculation must be statistically proven in further studies.

This study has limitations. First, the sample size was relatively small and no control group was present to thoroughly evaluate the effectiveness on the intervention. However, this study can have an important role in preliminary testing research instruments, verifying the usefulness of intervention methods, and pave the way for larger and final studies [63,64,65]. Second, we used self-report and report-form tools to evaluate maternal psychopathological risk and children’s emotional/behavioral problems, respectively. Despite the fact that the measures used were well validated and are extensively used in the literature, the information supplied may be impacted by perceptual biases and should be interpreted with caution. Finally, the sample’s homogeneity in terms of geographical origin does not allow broad generalization of the results to a wider population.

Notwithstanding the above limitations, this research bears several strengths. First, we used an observational measure to evaluate the quality of mother–child feeding interactions. International literature has largely demonstrated that observational tools constitute objective and informed clinical evaluation of the child’s behavioral and emotional characteristics [66]. Second, we considered the effects of the intervention on mothers’ and children’s sub-threshold difficulties, which are frequently overlooked by scientific literature and intervention programs. Third, very few examples of other web-based intervention strategies for feeding disorders in children have been proposed in previous literature [67,68]. Specifically, to the best of our knowledge, the present study is the first to focus on treatment based on online video-feedback subsequent to observational sessions of mother–child feeding interactions.

## 5. Conclusions

This article proposed the results of a study using an observational tool for the evaluation of the quality of parent–child interactions during feeding in a web-based context. This strategy was effective at enhancing the quality of parent–child exchanges, and maternal and children’s psychopathological symptoms during the COVID-19 lockdown, when in-person evaluation and treatment were hampered by the governments’ recommendations of maintaining interpersonal distancing and avoiding travelling. In fact, maternal and dyadic affective state, interactive conflict, and child’s food refusal, improved significantly, as well as offspring internalizing/externalizing problems and mothers’ depression, anxiety and obsession–compulsion symptoms. The results of this study showed that it is possible to effectively use this web-based method to achieve significant positive outcomes diminishing the costs of intervention.

## Figures and Tables

**Table 1:** Average scores and standard deviations of the SVIA subscales and general quality of mother–child feeding interactions.

	T1	T2	η^2^
	M (SD)	M (SD)	
Mother’s Affective State	22.73 (3.12)	12.12 (1.89) **	0.68
Interactive conflict	21.32 (3.21)	11.34 (2.45) **	0.48
Food Refusal Behavior	18.03 (2.91)	9.04 (1.68) **	0.56
Dyad’s Affective State	16.62 (2.31)	8.54 (1.24) **	0.69

η^2^: eta-squared. ** *p* < 0.001.

**Table 2:** Maternal scores at SCL-90/R.

	T1	T2	η^2^
	M (SD)	M (SD)	
SOM	0.29 (0.61)	0.21 (0.45)	0.17
O-C	0.61 (0.51)	0.22 (0.61) **	0.71
I-S	0.33 (0.42)	0.31 (0.33)	0.06
DEP	0.78 (0.24)	0.33 (0.69) **	0.79
ANX	0.61 (0.51)	0.23 (0.76) **	0.92
HOS	0.34 (0.41)	0.31 (0.54)	0.12
PHOB	0.12 (0.71)	0.18 (0.12)	0.16
PAR	0.35 (0.42)	0.31 (0.36)	0.18
PSY	0.19 (0.62)	0.14 (0.72)	0.15
GSI	0.89 (0.46)	0.51 (0.65) **	0.67

Note. SOM: Somatization; O-C: Obsessive–Compulsive; I-S: Interpersonal Sensitivity; DEP: Depression; ANX: Anxiety; HOS: Hostility; PHOB: Phobic Anxiety; PAR: Paranoid Ideation; PSY: Psychoticism. BSFC: Burden Scale for Family Caregivers. η^2^: eta-squared. ** *p* < 0.001.

**Table 3:** Means (standard deviation) of child’s CBCL subscales.

	T1	T2	η^2^
	M (SD)	M (SD)	
E-R	3.55 (1.51)	3.17 (2.37)	0.21
A-D	6.31 (1.45)	2.21 (1.62) **	0.81
S-C	3.65 (1.39)	4.01 (2.31)	0.18
WIT	7.14 (1.52)	3.43 (1.41) **	0.71
A-P	4.21 (1.37)	5.25 (1.21)	0.25
A-B	22.14 (4.25)	14.31 (3.18) **	0.68
INT	26.13 (2.61)	17.54 (3.14) **	0.64
EXR	25.43 (2.52)	16.61 (3.44) **	0.73

Note. E-R: Emotionally Reactive; A-D: Anxious/Depressed; S-C: Somatic Complaints; WIT: Withdrawn; A-P: Attention Problems; A-B: Aggressive Behavior; INT: Internalizing Problems; EXT: Externalizing Problems. η^2^: eta-squared. ** *p* < 0.001.

## Data Availability

The data presented in this study are openly available in FigShare at doi:10.6084/m9.figshare.14402444.
